# Immune Checkpoint Inhibitors in Glioblastoma IDHwt Treatment: A Systematic Review

**DOI:** 10.3390/cancers16244148

**Published:** 2024-12-12

**Authors:** Archit Bharathwaj Baskaran, Olivia A. Kozel, Omkar Venkatesh, Derek A. Wainwright, Adam M. Sonabend, Amy B. Heimberger, Rimas Vincas Lukas

**Affiliations:** 1 Department of Neurology, The University of Chicago, Chicago, IL 60637, USA; 2 Department of Neurosurgery, The Warren Alpert Medical School of Brown University, Providence, RI 02903, USA; olivia_kozel@brown.edu; 3 Feinberg School of Medicine, Northwestern University, Chicago, IL 60208, USA; omkar.venkatesh@gmail.com; 4 Departments of Cancer Biology & Neurological Surgery, Loyola University Chicago Stritch School of Medicine, Maywood, IL 60153, USA; dwainwr@luc.edu; 5 Department of Neurosurgery, Northwestern University, Chicago, IL 60208, USA; adam.sonabend@northwestern.edu (A.M.S.); amy.heimberger@northwestern.edu (A.B.H.); 6 Lou & Jean Malnati Brain Tumor Institute, Northwestern University, Chicago, IL 60611, USA; rimas.lukas@nm.org; 7 Department of Neurology, Northwestern University, Chicago, IL 60208, USA

**Keywords:** CTLA4, PD1, PD-L1, PD-L2, LAG3, glioblastoma, immune checkpoint inhibition

## Abstract

Purpose: A glioblastoma (GBM) is a primary brain tumor with significant unmet therapeutic needs. Immune checkpoint inhibitors (ICIs) have marked therapeutic benefits in many different cancers but have yet to show benefit for most GBM patients in phase III trials. Methods: A systematic review querying ClinicalTrials.gov for prospective clinical trials investigating ICI in GBM between 1950 and July 2024 was performed. Search terms comprised 11 distinct ICIs. Data abstracted include clinical trial NCT numbers with study titles and status, enrollment information, interventions, and more. Clinical trial identifying information, interventions, and outcomes were extracted. Results: One hundred and seventeen clinical trials were identified; four were phase 3. Most involved PD-1 or CTLA-4 blockade as monotherapy or in combination with standard-of-care. The large, randomized trials included CHECKMATE 143, CHECKMATE 498, CHECKMATE 548, and NRG BN007. These showed no improvement in median overall survival or progression-free survival in unselected patients. Biomarker-directed analyses suggest that a subset of GBM patients may benefit. Conclusions: ICI for the treatment of GBM has not demonstrated clear evidence of efficacy thus far. This review serves as a quick reference of ICI trial results in GBM. Biomarker-driven patient selection and/or novel approaches to overcome resistance mechanisms remain areas of viable inquiry.

## Simple Summary

We present a systematic review of the clinical trials of immune checkpoint inhibitors in GBM. This collates a substantial amount of data in a fashion that is manageable for the reader. It is anticipated that this will have value for the clinician managing this patient population, the clinical trialist contemplating the next therapeutic trials, and the scientist considering future investigations in immunotherapy.

## 1. Introduction

Glioblastoma IDH wild-type (GBM) is a primary central nervous system (CNS) tumor characterized by diffuse infiltration, high cellular proliferation, and heterogeneity of genomic features. Fc-enhanced anti-CTLA-4, anti-PD-1, doxorubicin, and ultrasound-mediated BBB opening are novel combinatorial immunotherapy regimens for gliomas. The current standard of care involves maximum safe surgical resection, radiation, temozolomide, and tumor-treating fields in the newly diagnosed setting [1,2]. In progressive disease, the optimal management is less clearly defined, but the nitrosourea CCNU is commonly used [3].

We review the mechanisms whereby immune checkpoints regulate and impede anti-tumor immune responses. This is followed by a systematic review of the clinical trials in which immune checkpoint inhibitors (ICIs) have been evaluated in patients with a GBM that predominantly focused on cytotoxic T lymphocyte-associated protein 4 (CTLA-4) and programmed death 1 (PD-1). A comprehensive understanding of what has been clinically evaluated informs rational decision-making on future strategies. Finally, we briefly review potential mechanisms that have impeded the success of ICIs for the treatment of GBMs and biomarkers that may be selected for responsive patients.

### 1.1. T Cell Activation and Inhibitory Pathways

T cells contribute to the primary adaptive anti-tumor response. Antigen-presenting cells (APCs) present antigens on major histocompatibility complexes (MHCs) located on the cell surface. Broadly, MHC class I (MHC I) presents endogenous peptides, while MHC class II (MHC II) presents antigens phagocytosed by the APCs. When an APC presents an antigen on the MHC complex that has sufficient affinity for a T cell receptor (TCR), additional signals determine the activation status of the T cell [4]. Co-stimulation of T cells can lead to cytokine release and cytotoxicity; however, insufficient co-stimulation or expression of immune checkpoints triggers T cell anergy. With subsequent rounds of stimulation, immune checkpoints become upregulated to generate a state of immune exhaustion that down-regulates immune responses. CTLA-4 on the T cell signals through the immunoreceptor tyrosine-based inhibitory motif domain when it binds to B7 on other cells, thereby suppressing function [5]. The endogenous function of PD-1 is to act as a negative regulator of the immune response by binding to its ligands, PD-L1 or PD-L2, which are expressed on various cells, including cancer cells, normal non-immune cells, and some immune cells, including dendritic cells and B-cells. This binding leads to the inhibition of T cell activation and proliferation. T cell exhaustion is a state in which anti-tumor effector responses cannot be re-invigorated with ICI and is particularly problematic in GBM [6]. Blocking CTLA-4 and/or PD-1 increases net T cell activation [7]. There have been several pivotal trials that have demonstrated the efficacy of ICIs by improving overall survival (OS), progression-free survival (PFS), and landmark survival in the treatment of non-CNS cancers [8,9,10,11,12,13,14,15].

**Figure 1:**
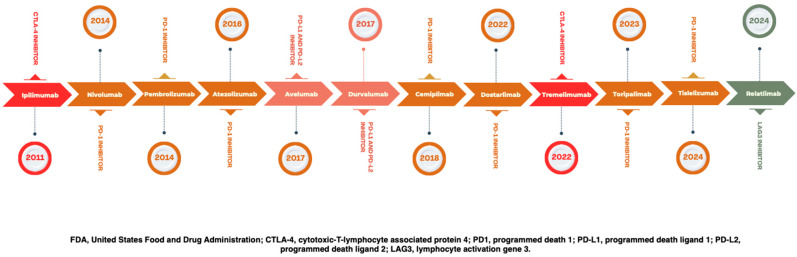
Immune checkpoint inhibitor molecular targets and FDA approval timeline.

An overview of current Food and Drug Administration (FDA)-approved ICIs within the United States is displayed in Figure 1. In 2011, ipilimumab became the first CTLA-4 inhibiting drug to receive regulatory approval [16]. Many other ICIs subsequently followed. More recently, other checkpoints have gained interest, including lymphocyte activation gene 3 (LAG-3) [17], which is targeted with relatlimab and is now approved to treat advanced melanoma in combination with nivolumab [18]. This checkpoint, however, has limited expression in GBMs and is even less frequently expressed in lower-grade infiltrating gliomas [19].

### 1.2. Role of Myeloid Cells in GBM Tumor Microenvironment

The role of myeloid cells, such as macrophages, is central to the tumor microenvironment. Even compared to T cells, as mentioned prior, myeloid cells may play a more prominent role in GBMs. Their role is more extensively elaborated upon in the Discussion section of this paper.

## 2. Materials and Methods

A systematic review was conducted utilizing ClinicalTrials.gov and queried for all relevant clinical trials corresponding to the designated search terms. Search terms were limited to therapeutic agents with regulatory approval in the United States. None of these agents have regulatory approval for GBM. All studies published between 1950 and April 2024 were screened, and applicable studies underwent data abstraction and analysis. Unique searches were run for each of the following terms corresponding to the appropriate ICI, with the search yielding the following number of articles:

1. “nivolumab “AND “glioblastoma”—39 articles;
2. “pembrolizumab” AND “glioblastoma”—38 articles;
3. “ipilimumab” AND “glioblastoma”—19 articles;
4. “avelumab” AND “glioblastoma”—5 articles;
5. “durvalumab” AND “glioblastoma”—4 articles;
6. “tislelizumab” AND “glioblastoma”—4 articles;
7. “cemiplimab” AND “glioblastoma”—3 articles;
8. “tremelimumab” AND “glioblastoma”—1 article;
9. “dostarlimab” AND “glioblastoma”—0 articles;
10. “toripalimab” AND “glioblastoma”—0 articles;
11. “relatlimab” AND “glioblastoma”—3 articles.

The data were extracted by three independent reviewers (AB, OK, and OV) who conducted individual searches on clinicaltrials.gov using the search strategy described above. After completing the search, the authors convened to discuss whether the identified studies met the inclusion criteria. The criteria were as follows: (1) articles published after 1950, (2) studies involving patients diagnosed with glioblastoma multiforme, and (3) trials involving FDA-approved immune checkpoint inhibitors. Initial trials were retrieved for further analysis, as illustrated in the flowchart in Figure 1. RL subsequently reviewed and independently verified that all articles met the inclusion criteria, performing an additional quality check to ensure that all identified clinical trials involved immune checkpoint inhibitors currently used in clinical practice. The search strategy, along with the number of relevant articles identified through each search term, is outlined in the Section 2. Of note, each study had an assigned designation, such as active, recruiting, not recruiting, etc. Some studies were designated withdrawn or suspended for a variety of reasons, including lack of patient recruitment or sufficient funding.

**Figure 2:**
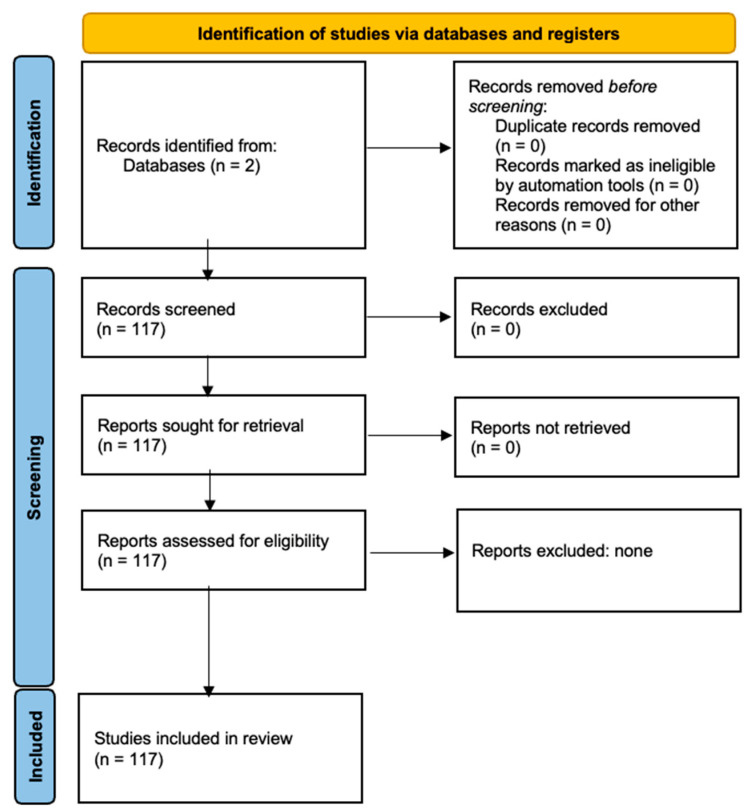
PRISMA flow diagram for systematic reviews, which included searches of databases and registers only.

Data abstracted from individual articles included clinical trial identifying information such as NCT numbers, study titles, study status, agents investigated, and conditions investigated that were filtered by ‘glioblastoma’, interventions, study phase, enrollment population, and start date. Additional information abstracted by reviewers included study sponsor, primary outcomes, and secondary outcomes data where relevant, along with molecular mechanisms of various agents utilized in the clinical trials. We have created and included a flow chart detailing our methodology for study selection. Please see the flowchart in Figure 2.

## 3. Results

In total, the search yielded 117 clinical trials. They are compiled in Table 1. Following this initial search, an additional search was performed to inspect the descriptions or bibliographies of articles for other studies, and none were identified. All 117 articles represented relevant clinical trials.

Among the late-stage clinical trials identified, one was phase 2/3, and three were phase 3, of which progression-free survival (PFS) and median overall survival (mOS) are compiled in Table 2.

### Limitations

Limitations include the fact that the search strategy was limited to agents with U.S. FDA regulatory approval for cancer. This allowed a focus on agents extensively studied for safety and have demonstrated efficacy in other malignancies. If efficacious, these ICIs could be rapidly implemented for routine use in GBM. This narrow focus avoids the pitfall of including less extensively studied ICI while inadvertently not including others, creating an imbalanced perspective. Nonetheless, such an approach has the potential to highlight agents with substantial promise for patients with GBM.

## 4. Discussion

### 4.1. Summary of Clinical Outcomes in GBM

Several studies have examined the role of ICIs in the treatment of GBM. The majority of these have focused on PD-1 blockade in both the newly diagnosed and recurrent settings. Treatment ranged from monotherapy to multi-modality regimens incorporating surgery, radiation therapy, chemotherapy, tumor treating fields, and other immunotherapies.

The largest of these studies utilized the PD-1 antibody, nivolumab, with or without the CTLA-4 antibody, ipilimumab. CHECKMATE 143 was a multi-arm randomized phase 3 trial with cohorts for newly diagnosed and recurrent GBM. A variety of therapeutic regimens were investigated. One cohort for recurrent GBM compared nivolumab vs. bevacizumab, but the mOS was similar (9.8 vs. 10.0 months, hazard ratio (HR) = 1.04, *p* = 0.76) [115]. Another smaller cohort (*n* = 40) of recurrent GBM patients compared nivolumab (3 mg/kg) vs. nivolumab (1 mg/kg) + ipilimumab (3 mg/kg) and found the combination to have worse tolerability. A non-randomized cohort using alternate dosing of nivolumab (3 mg/kg) + ipilimumab (1 mg/kg) was found to be better tolerated [116] and influenced the subsequent dosing regimen of the combination in the NRG BN007 trial. This same CHECKMATE 143 trial also included a large (*n* = 136) exploratory phase 1 component evaluating nivolumab + radiotherapy +/− temozolomide in newly diagnosed GBM. This cohort demonstrated no new safety signals and promising OS in the various subgroups [116], leading to further exploration of ICI-based approaches in two definitive phase 3 trials for newly diagnosed GBM. CHECKMATE 548 evaluated the efficacy of nivolumab, radiation, and temozolomide in MGMT promoter methylated newly diagnosed GBM [117]. Simultaneously, CHECKMATE 498 evaluated the efficacy of nivolumab and radiation (omitting temozolomide from the regimen) in MGMT promoter unmethylated newly diagnosed GBM [118]. Both large, randomized trials were negative without significant improvement in OS or PFS relative to standard of care. Neither study allowed for tumor-treating fields, which had previously been shown to improve OS when added to standard-of-care, and for which there are suggestions of possible synergy with ICI [119,120]. The NRG BN007 phase 2/3 trial evaluated the combination of nivolumab and ipilimumab using the better-tolerated dosing regimen explored in CHECKMATE 143 and allowing the use of tumor-treating fields at the treating physicians’ discretion in newly diagnosed MGMT promoter unmethylated GBM. However, this study also showed no improvement PFS after completion of the phase 2 component [121]. OS data are still pending at this time of manuscript completion.

There is an incomplete understanding of the prevalence of the aforementioned markers’ prevalence of expression (for example, PD-1, PD-L1, CTLA-4, and ligands CD80 or CD86) in different GBM stages or subtypes (as defined by methylation class), be it newly diagnosed or recurrent/progressive, or after specific treatments (post-radiation vs. post-chemotherapy). This is due to a combination of the dynamic expression of these checkpoints and the difficulty in obtaining repeated tumor tissue samples (particularly when not clinically indicated). However, it has been shown that expression of the markers such as PD-L1 can vary broadly (0–87% of cells in human GBM samples) with a median near ~3% (viewed as “positive” PD-L1 expression in other cancers). PD1 and PD-L1 expression in human gliomas appears to be predominantly present on CD8+ and CD4+ T cells, with minimal expression on glioma cells [122]. In turn, it is unsurprising that the overall PD1/PDL1 expression in these tumors is low as lymphocytes comprise only a very small portion of the tumor mass.

Furthermore, upon further examination, it is not reliably reported for each study how many patients with IDH wt GBM specifically were included in each trial. This is related to the differing inclusion/exclusion criteria for each study. For example, one trial may specify “initial diagnosis of unmethylated glioblastoma”, and another may simply list “stage IV glioblastoma” without further detail. Therefore, we are not able to decisively report this for all studies in Table 1.

### 4.2. Potential Predictive Biomarkers

There may be subsets of patients who selectively benefit from ICI. Considering several negative trials, it will be difficult to justify embarking on similarly sized studies in select subgroups. Nonetheless, biomarker studies may help inform how immunotherapeutic approaches could be advanced in GBM patients. Potential predictive biomarkers assessed for ICI benefit in GBM patients include tumor PD-L1/2 expression, CTLA-4 expression, mismatch repair deficiency (MMRd), tumor mutation burden (TMB), tumor-infiltrating lymphocytes, tumor-specific antibodies, and T cell functional markers [123]. In contrast to other malignancies, TMB is not a predictor of response to ICI in GBM [124]. Thus far, no biomarker has been prospectively validated in this setting.

Since standard immune biomarkers have not been useful in GBM, researchers have searched for other indicators to identify responses to immunotherapy. In one such example, we reported longitudinal genomic and transcriptomic analysis of recurrent GBM patients, including long-term responders. Our group found that non-responders were significantly enriched for immunosuppressive phosphatase and tensin homolog (PTEN) mutations, while responders were more likely to have mitogen-activated protein kinase (MAPK) pathway aberrancies including protein tyrosine phosphatase non-receptor type 11 (PTPN11) and B rapidly accelerated fibrosarcoma (BRAF) mutations [125]. Responders had greater T cell infiltration, and there was evidence of selection against neoepitopes in responders. Our subsequent study attempted to interrogate the mechanism by which BRAF and PTPN11 mutations might promote a response to ICIs in recurrent GBM [125]. We hypothesized that activation of MAP/ERK signaling downstream of BRAF and PTPN11 would be associated with an improved response to a PD-1 inhibitor. Immunohistochemistry for phosphorylated ERK1/2 (p-ERK) was predictive of OS in recurrent GBM patients treated with adjuvant PD-1 inhibition in two separate independent patient cohorts. sc-RNA-seq showed that p-ERK localized to tumor cells with an associated robust microglial infiltration, which exhibited antigen-presenting phenotype, that the investigators hypothesized contributes to the favorable response [126]. More recently, a Reporting Recommendations for Tumor Marker Prognostic Studies (REMARK)-criteria blinded analysis of p-ERK established that this biomarker predicted OS from a trial in which recurrent GBM patients underwent administration of cavitary and systemic anti-CTLA-4 and anti-PD1 treatments [127]. Previously, using murine models, our same group revealed that if glioma formation takes place in the absence of T cells, MAPK becomes activated in the resulting tumors, supporting that this signaling cascade promotes tumor immunogenicity [128].

The aforementioned biomarkers could be leveraged in future clinical trials to enable more optimal personalized immunotherapeutic approaches and outcomes for patients with GBM. For example, prospective use of markers such as MAPK pathway activation (such as pERK) MMRd, CTLA-4, and PD-L1 or PD-L2 expression in clinical trials may help elucidate which patients may benefit from different ICI agents alone or as components of combinatorial regimens. It should be reinforced, however, that to date, prospective studies have yet to validate any single biomarker in this context, as noted previously. However, the incorporation of biomarkers in future trials remains a valuable area of investigation.

### 4.3. Neoadjuvant Monotherapy

In addition to patient selection, therapeutic timing may be a criterion for benefit. Two small studies have evaluated the role of ICIs in the neoadjuvant setting. One compared neoadjuvant and adjuvant pembrolizumab vs. adjuvant pembrolizumab monotherapy and showed a survival benefit in the neoadjuvant arm with a mOS of 14 months vs. 7.5 in the adjuvant-only arm. The results were hypothesized to be mediated by IFN-γ-mediated T cell activation based on the results of single-cell RNA sequencing of tissue [129]. These findings may be due to imbalances in STING expression between the two arms. The second study from de Groot et al. studied therapeutic targeting with pembrolizumab monotherapy in GBM patients during a set “window-of-opportunity” for intervention, ultimately concluding that despite timely treatment, PD-1 monotherapy alone is not sufficient to mount an efficient anti-tumor immunologic response [130].

### 4.4. Strategies for Reprogramming the GBM Tumor Microenvironment

GBM utilizes multiple redundant mechanisms of immune evasion to develop and thrive. These include the promotion of a relatively immunologically cold microenvironment, alteration of the peripheral immune system via lymphosuppression, genomic heterogeneity, and the blood-brain barrier (BBB) [131]. Immune checkpoint expression is one of several mechanisms for how GBM evades the immune system and contributes to the lack of success with ICI.

T cell exhaustion is characterized by the upregulation of multiple immune checkpoints and is not reversible [132]. Exhaustion is a significant mode of T cell dysfunction across cancers, especially in GBM, and highlights the need to address underlying mechanisms that contribute to tumor-imposed exhaustion to formulate effective immunotherapies [133]. Beyond direct effects on the T cells, other elements such as sequestration of the T cells outside the CNS [134], diffuse immunosuppression throughout the CNS, which worsens with aging or treatments such as radiation therapy [135,136], and an overabundance of myeloid-derived immune cells with immunosuppressive polarization may serve as potential therapeutic targets.

Najem et al. studied the neoadjuvant STING agonist 8803 in multiple preclinical models, first as monotherapy in ICI-resistant models of mice with QPP8 tumors and second as combination therapy with STAT3 inhibitors or PD-1 inhibitors [137]. The 8803 molecule was administered directly into the GBM to circumvent the blood-brain barrier. In the study, 100% of mice with QPP8 tumors treated with 8803 were cured, demonstrating increased median overall survival. Mice treated with combination 8803 therapy with anti-PD-1 blockade demonstrated increased survival. In contrast, 8803 combination therapy with STAT3 inhibitors did not amplify the effects of STING agonism [137]. This may be unsurprising as STING agonism and STAAT3 inhibition can be viewed as complementary components of the same immune mechanism. This is somewhat analogous to a lack of additive benefit from combining PD-1 and PD-L1 blockade. Altogether, these findings demonstrate the potential for clinical translation of STING agonism in combination with PD-1/PD-L1 blockade in preclinical models and clinical trials.

### 4.5. Myeloid Cells

Myeloid cells, including macrophages and microglia, play a role in the immune evasion of GBM. Their role in creating an immunosuppressive tumor microenvironment in GBMs is of substantial importance and contributes to the attenuation of ICI.5 This may help to explain the results observed in many of the trials discussed above. Microglia/macrophages are the predominant type of immune cells that infiltrate GBM, and their capacity to be stimulated and activate antitumor effector T cells is not sufficient to initiate immune responses [138]. These myeloid cells are also a prominent type of immune cell that accounts for up to 50% of total cells in GBM, and their context-dependent interactions within GBM are pivotal for tumor growth and progression. Macrophage infiltration is closely correlated with vascular density in human gliomas [139]. These may prove to be a valid target in combinatorial regimens, which also either provide direct tumor cell cytotoxicity or immune stimulation in addition to abrogation of the immunosuppressive microenvironment.

Several groups have reported that a small dose of doxorubicin can have immune-modulating properties, activate the STING pathway [140,141,142], and enhance the response of cancer to ICI. Indeed, a recent clinical trial showed that compared to other induction regimens, low-dose doxorubicin doubled the response rate of breast cancer patients to anti-PD1 [143]. Doxorubicin as an immune modulator has also been studied in GBM, including a recent report where we showed that in a cohort of GBM treated with doxorubicin, anti-PD1 ICI, and ultrasound-based blood-brain barrier opening, doxorubicin promoted the expression of MHC antigen-presenting molecules the tumor cells tumor-associated microglia and IFN-gamma expression by microglia infiltrating T cells [144].

## 5. Conclusions

ICI has demonstrated success across a variety of malignancies. To date, similar results have not been observed in patients with GBM. An understanding of previous clinical investigations and their results, paired with an understanding of correlative and preclinical studies, can help guide the next steps in the investigation. Future approaches that use ICI in high-grade glioma may focus on using these agents as an adjunct in mechanistically rational combinations. Due to the well-understood mechanism of action, reasonable safety profile, and substantial experience with these agents in the neuro-oncology field, ICI may be easily incorporated into investigations of other therapeutic treatment approaches for glioblastoma.

## Figures and Tables

**Table 1:** Compilation of the agent(s) investigated with their identifying clinical trial number, phase, enrollment, study status, and timeline information.

Agent Name	NCT Number	Study Title	Study Status	Interventions	Phase	Enrollment	Start Date
Nivolumab	NCT02017717	A Study of the Effectiveness and Safety of Nivolumab Compared to Bevacizumab and of Nivolumab with or Without Ipilimumab in Glioblastoma Patients [20]	Active (not recruiting)	Nivolumab, Bevacizumab, Ipilimumab	3	529	7 February 2014
Nivolumab	NCT02327078	A Study of the Safety, Tolerability, and Efficacy of Epacadostat Administered in Combination with Nivolumab in Select Advanced Cancers (ECHO-204) [21]	Completed	Nivolumab, Epacadostat	1/2	307	26 November 2014
Nivolumab	NCT02335918	A Dose Escalation and Cohort Expansion Study of Anti-CD27 (Varlilumab) and Anti-PD-1 (Nivolumab) in Advanced Refractory Solid Tumors [22]	Completed	Varlilumab, Nivolumab	1/2	175	January 2015
Nivolumab	NCT02311920	Ipilimumab and/or Nivolumab in Combination with Temozolomide in Treating Patients with Newly Diagnosed Glioblastoma or Gliosarcoma [23]	Completed	Ipilimumab, Nivolumab, Temozolomide	1	32	16 April 2015
Nivolumab	NCT02550249	Neoadjuvant Nivolumab in Glioblastoma [24]	Completed	Nivolumab	2	29	June 2015
Nivolumab	NCT02529072	Nivolumab With DC Vaccines for Recurrent Brain Tumors [25]	Completed	Nivolumab, Dendritic cells	1	6	January 2016
Nivolumab	NCT02617589	CHECKMATE 498: An Investigational Immuno-Therapy Study of Nivolumab Compared to Temozolomide, Each Given With Radiation Therapy, for Newly-diagnosed Patients With Glioblastoma (GBM, a Malignant Brain Cancer) [26]	Completed	Nivolumab, Temozolomide, Radiotherapy	3	560	1 March 2016
Nivolumab	NCT02667587	CHECKMATE 548: An Investigational Immuno-Therapy Study of Temozolomide Plus Radiation Therapy With Nivolumab or Placebo, for Newly Diagnosed Patients With Glioblastoma (GBM, a Malignant Brain Cancer) [27]	Active (not recruiting)	Nivolumab, Temozolomide, Radiotherapy, Nivolumab Placebo	3	716	9 May 2016
Nivolumab	NCT02648633	Stereotactic Radiosurgery with Nivolumab and Valproate in Patients with Recurrent Glioblastoma [28]	Terminated	Stereotactic Radiosurgery, Nivolumab, Valproate	1	4	24 May 2016
Nivolumab	NCT02658981	Anti-LAG-3 Alone and in Combination with Nivolumab Treating Patients with Recurrent GBM (Anti-CD137 Arm Closed 10/16/18) [29]	Completed	Anti-LAG-3 Monoclonal Antibody BMS 986016, Nivolumab, Anti-CD137	1	63	24 August 2016
Nivolumab	NCT03233152	Intra-tumoral Ipilimumab Plus Intravenous Nivolumab Following the Resection of Recurrent Glioblastoma [30]	Unknown	Ipilimumab, Nivolumab	1	110	17 November 2016
Nivolumab	NCT03879512	Autologous Dendritic Cells, Metronomic Cyclophosphamide and Checkpoint Blockade in Children with Relapsed HGG [31]	Recruiting	Dendritic cells, Cyclophosphamide, Nivolumab	1/2	25	7 February 2018
Nivolumab	NCT03367715	Nivolumab, Ipilimumab, and Short-course Radiotherapy in Adults with Newly Diagnosed, MGMT Unmethylated Glioblastoma [32]	Completed	Nivolumab, Ipilimumab, Radiation Therapy	2	10	7 February 2018
Nivolumab	NCT04195139	Nivolumab and Temozolomide Versus Temozolomide Alone in Newly Diagnosed Elderly Patients with GBM [33]	Active (not recruiting)	Nivolumab, Temozolomide	2	103	22 February 2018
Nivolumab	NCT03576612	GMCI, Nivolumab, and Radiation Therapy in Treating Patients with Newly Diagnosed High-Grade Gliomas [34]	Active (not recruiting)	AdV-tk, Valacyclovir, Radiation Therapy, Temozolomide, Nivolumab	1	36	27 February 2018
Nivolumab	NCT03452579	Nivolumab Plus Standard Dose Bevacizumab Versus Nivolumab Plus Low Dose Bevacizumab in GBM [35]	Active (not recruiting)	Nivolumab, Bevacizumab	2	90	21 May 2018
Nivolumab	NCT03636477	A Study of Ad-RTS-hIL-12 With Veledimex in Combination with Nivolumab in Subjects With Glioblastoma; a Substudy to ATI001-102 [36]	Completed	Ad-RTS-hIL-12, Veledimex, Nivolumab	1	21	18 June 2018
Nivolumab	NCT03493932	Cytokine Microdialysis for Real-Time Immune Monitoring in Glioblastoma Patients Undergoing Checkpoint Blockade [37]	Completed	Nivolumab, BMS-986016	1	21	24 September 2018
Nivolumab	NCT03890952	Translational Study of Nivolumab in Combination with Bevacizumab for Recurrent Glioblastoma [38]	Active (not recruiting)	Nivolumab, Bevacizumab	2	40	1 October 2018
Nivolumab	NCT03422094	Neoantigen-based Personalized Vaccine Combined with Immune Checkpoint Blockade Therapy in Patients with Newly Diagnosed, Unmethylated Glioblastoma [39]	Terminated	NeoVax, Nivolumab, Ipilimumab	1	3	31 October 2018
Nivolumab	NCT03684811	A Study of FT-2102 in Patients with Advanced Solid Tumors and Gliomas With an IDH1 Mutation [40]	Completed	FT-2102, Azacitidine, Nivolumab, Gemcitabine, Cisplatin	1/2	93	1 November 2018
Nivolumab	NCT03743662	Nivolumab With Radiation Therapy and Bevacizumab for Recurrent MGMT Methylated Glioblastoma [41]	Active (not recruiting)	Radiation therapy, Bevacizumab, Nivolumab	2	39	12 November 2018
Nivolumab	NCT03430791	Trial of Combination Tumor Treating Fields (TTF; Optune), Nivolumab Plus/Minus Ipilimumab for Recurrent Glioblastoma [42]	Terminated	Nivolumab, Ipilimumab, NovoTTF200A (Optune)	2	5	5 December 2018
Nivolumab	NCT03707457	Biomarker-Driven Therapy Using Immune Activators with Nivolumab in Patients with First Recurrence of Glioblastoma [43]	Terminated	Nivolumab, Anti-GITR Monoclonal Antibody MK-4166, IDO1 inhibitor INCB024360, Ipilimumab	1	3	22 March 2019
Nivolumab	NCT03718767	Nivolumab in Patients With IDH-Mutant Gliomas with and Without Hypermutator Phenotype [44]	Recruiting	Nivolumab	2	70	27 March 2019
Nivolumab	NCT04047706	Nivolumab, BMS-986205, and Radiation Therapy With or Without Temozolomide in Treating Patients with Newly Diagnosed Glioblastoma [45]	Active (not recruiting)	IDO1 Inhibitor BMS-986205, Nivolumab, Radiation Therapy, Temozolomide	1	18	13 August 2019
Nivolumab	NCT03014804	Autologous Dendritic Cells Pulsed with Tumor Lysate Antigen Vaccine and Nivolumab in Treating Patients with Recurrent Glioblastoma [46]	Withdrawn	Dendritic cells, Nivolumab	2	0	1 December 2019
Nivolumab	NCT04003649	IL13Ra2-CAR T Cells with or Without Nivolumab and Ipilimumab in Treating Patients with GBM [47]	Recruiting	IL13Ralpha2-CAR T cell, Ipilimumab, Nivolumab	1	60	2 December 2019
Nivolumab	NCT04116658	First-in-Human, Phase 1b/2a Trial of a Multipeptide Therapeutic Vaccine in Patients with Progressive Glioblastoma [48]	Active (not recruiting)	EO2401, Nivolumab	1/2	100	13 July 2020
Nivolumab	NCT04396860	Testing the Use of the Immunotherapy Drugs Ipilimumab and Nivolumab Plus Radiation Therapy Compared to the Usual Treatment (Temozolomide and Radiation Therapy) for Newly Diagnosed MGMT Unmethylated Glioblastoma [49]	Active (not recruiting)	Ipilimumab, Nivolumab, NovoTTF-100A, Radiation Therapy, Temozolomide	2/3	147	1 September 2020
Nivolumab	NCT04323046	Immunotherapy Before and After Surgery for Treatment of Recurrent or Progressive High-Grade Glioma in Children and Young Adults [50]	Recruiting	Nivolumab	1	20	2 October 2020
Nivolumab	NCT04145115	A Study Testing the Effect of Immunotherapy (Ipilimumab and Nivolumab) in Patients with Recurrent Glioma with Elevated Mutational Burden [51]	Suspended	Ipilimumab, Nivolumab	2	37	24 December 2020
Nivolumab	NCT04606316	Surgical Nivolumab and Ipilimumab for Recurrent GBM [52]	Active (not recruiting)	Nivolumab, Placebo, Ipilimumab	1	63	1 February 2021
Nivolumab	NCT04704154	A Trial to Learn Whether Regorafenib in Combination with Nivolumab Can Improve Tumor Responses and How Safe it Is for Participants with Solid Tumors [53]	Active (not recruiting)	Regorafenib (Stivarga, BAY73-4506), Nivolumab	2	175	3 February 2021
Nivolumab	NCT04817254	Association of Peripheral Blood Immunologic Response to Therapeutic Response to Adjuvant Treatment with Immune Checkpoint Inhibition (ICI) in Patients with Newly Diagnosed Glioblastoma or Gliosarcoma [54]	Recruiting	Ipilimumab, Nivolumab	2	58	8 December 2021
Nivolumab	NCT05909618	Crizanlizumab Alone or in Combination with Nivolumab for Glioblastoma and Melanoma with Brain Metastases [55]	Recruiting	Crizanlizumab, Nivolumab	2	33	11 July 2023
Nivolumab	NCT06047379	Safety and Efficacy of NEO212 in Patients with Astrocytoma IDH-mutant, Glioblastoma IDH-wildtype or Brain Metastasis [56]	Recruiting	NEO212, Ipilimumab, Pembrolizumab, Nivolumab. Regorafenib, Carboplatin, Paclitaxel, FOLFIRI, Bevacizumab	1/2	134	1 November 2023
Nivolumab	NCT06097975	A Clinical Trial on Combined (Neo-)Adjuvant Intravenous Plus Intracranial Administration of Ipilimumab and Nivolumab in Recurrent Glioblastoma [57]	Not yet recruiting	Nivolumab, Ipilimumab	1	18	1 January 2024
Nivolumab	NCT06325683	Anti-Lag-3 (Relatlinib) and Anti-PD-1 Blockade (Nivolumab) Versus Standard of Care (Lomustine) for the Treatment of Patients with Recurrent Glioblastoma [58]	Not yet recruiting	Anti-Lag-3 (Relatlinib), Lomustine, Nivolumab	2	178	21 June 2024
Pembrolizumab	NCT02430363	Evaluation Of the Treatment Effectiveness Of Glioblastoma / Gliosarcoma Through The Suppression Of The PI3K/Akt Pathway Compared With MK-3475 [59]	Unknown	Pembrolizumab, suppressors of the PI3K/Akt pathways	1/2	58	1 March 2013
Pembrolizumab	NCT02287428	Personalized Neoantigen Cancer Vaccine with Radiation Therapy Plus Pembrolizumab for Patients With Newly Diagnosed GBM [60]	Recruiting	Radiation Therapy, Neoantigen Vaccine, Pembrolizumab, Temozolomide	1	56	1 November 2014
Pembrolizumab	NCT02337491	Pembrolizumab with or without evacizumab for Recurrent Glioblastoma Multiforme [61]	Completed	Pembrolizumab, Bevacizumab	2	80	9 February 2015
Pembrolizumab	NCT02337686	Pembrolizumab in Treating Patients with Recurrent Glioblastoma [62]	Active (not recruiting)	Pembrolizumab	2	18	28 April 2015
Pembrolizumab	NCT02530502	Radiation Therapy with Temozolomide and Pembrolizumab in Treating Patients with Newly Diagnosed Glioblastoma [63]	Terminated	Pembrolizumab, Radiation Therapy, Temozolomide	1	4	30 September 2015
Pembrolizumab	NCT02852655	A Pilot Surgical Trial to Evaluate Early Immunologic Pharmacodynamic Parameters For The PD-1 Checkpoint Inhibitor, Pembrolizumab (MK-3475), In Patients With Surgically Accessible Recurrent/Progressive Glioblastoma [64]	Completed	Pembrolizumab	1	25	21 September 2016
Pembrolizumab	NCT02798406	Combination Adenovirus + Pembrolizumab to Trigger Immune Virus Effects [65]	Completed	DNX-2401, Pembrolizumab	2	49	6 October 2016
Pembrolizumab	NCT03197506	Pembrolizumab and Standard Therapy in Treating Patients with Glioblastoma [66]	Suspended	Radiation Therapy, Pembrolizumab, Temozolomide	2	52	15 September 2017
Pembrolizumab	NCT03018288	Radiation Therapy Plus Temozolomide and Pembrolizumab with and Without HSPPC-96 in Newly Diagnosed Glioblastoma (GBM) [67]	Completed	Pembrolizumab, HSPPC-96, Temozolomide	2	90	21 September 2017
Pembrolizumab	NCT03277638	Laser Interstitial Thermotherapy (LITT) Combined with Checkpoint Inhibitor for Recurrent GBM (RGBM) [68]	Recruiting	Pembrolizumab, Laser Interstitial Thermotherapy	1/2	34	29 November 2017
Pembrolizumab	NCT03347617	Ferumoxytol MRI in Assessing Response to Pembrolizumab in Patients with Glioblastoma [69]	Active (not recruiting)	Ferumoxytol, Pembrolizumab	2	56	20 December 2017
Pembrolizumab	NCT03405792	Study Testing the Safety and Efficacy of Adjuvant Temozolomide Plus Tumor Treating Fields (TTF) (Optune) Plus Pembrolizumab in Patients with Newly Diagnosed Glioblastoma (2-THE-TOP) [70]	Active (not recruiting)	Temozolomide, Optune, Pembrolizumab	2	40	23 February 2018
Pembrolizumab	NCT03426891	Pembrolizumab and Vorinostat Combined with Temozolomide for Newly Diagnosed Glioblastoma [71]	Completed	Pembrolizumab, Vorinostat, Temozolomide, Radiation therapy	1	21	16 March 2018
Pembrolizumab	NCT03661723	Pembrolizumab and Reirradiation in Bevacizumab Naive and Bevacizumab Resistant Recurrent Glioblastoma [72]	Active (not recruiting)	Pembrolizumab, Bevacizumab, Radiation therapy	2	60	28 September 2018
Pembrolizumab	NCT03665545	Pembrolizumab in Association with the IMA950/Poly-ICLC for Relapsing Glioblastoma [73]	Active (not recruiting)	IMA950, Poly-ICLC, Pembrolizumab	1/2	18	25 October 2018
Pembrolizumab	NCT03722342	TTAC-0001 and Pembrolizumab Combination phase1b Trial in Recurrent Glioblastoma [74]	Active (not recruiting)	TTAC-0001, Pembrolizumab	1	9	16 January 2019
Pembrolizumab	NCT03797326	Efficacy and Safety of Pembrolizumab (MK-3475) Plus Lenvatinib (E7080/MK-7902) in Previously Treated Participants with Select Solid Tumors (MK-7902-005/E7080-G000-224/LEAP-005) [75]	Active (not recruiting)	Pembrolizumab, Lenvatinib	2	590	12 February 2019
Pembrolizumab	NCT03726515	CART-EGFRvIII + Pembrolizumab in GBM [76]	Completed	EGFRvIII T CAR T cells, Pembrolizumab	1	7	11 March 2019
Pembrolizumab	NCT04121455	Glioblastoma Treatment with Irradiation and Olaptesed Pegol (NOX-A12) in MGMT Unmethylated Patients [77]	Active (not recruiting)	Olaptesed pegol, Radiation Therapy, Bevacizumab. Pembrolizumab	1/2	27	12 September 2019
Pembrolizumab	NCT03951142	Imaging Perfusion Restrictions from Extracellular Solid Stress—An Open-label Losartan Study [78]	Enrolling by invitation	Losartan, Pembrolizumab	2	165	1 October 2019
Pembrolizumab	NCT04201873	Pembrolizumab and a Vaccine (ATL-DC) for the Treatment of Surgically Accessible Recurrent Glioblastoma [79]	Recruiting	Dendritic Cells, Pembrolizumab, Poly ICLC	1	40	8 January 2020
Pembrolizumab	NCT04013672	Study of Pembrolizumab Plus SurVaxM for Glioblastoma at First Recurrence [80]	Active (not recruiting)	Pembrolizumab, SurVaxM, Sargramostim, Montanide ISA 51	2	40	19 March 2020
Pembrolizumab	NCT03899857	Pembrolizumab for Newly Diagnosed Glioblastoma [81]	Active (not recruiting)	Pembrolizumab	2	56	21 October 2020
Pembrolizumab	NCT04479241	LUMINOS-101: Lerapolturev (PVSRIPO) and Pembrolizumab in Patients with Recurrent Glioblastoma [82]	Active (not recruiting)	Lerapolturev, Pembrolizumab	2	30	21 October 2020
Pembrolizumab	NCT04913337	Study of NGM707 as Monotherapy and in Combination with Pembrolizumab in Advanced or Metastatic Solid Tumor Malignancies [83]	Recruiting	NGM707, Pembrolizumab	1/2	179	9 June 2021
Pembrolizumab	NCT05053880	A Study to Evaluate Safety and Efficacy of ACT001 and Anti-PD-1 in Patients with Surgically Accessible Recurrent Glioblastoma Multiforme [84]	Unknown	ACT001, Pembrolizumab	1/2	48	22 September 2021
Pembrolizumab	NCT04118036	Abemaciclib + Pembrolizumab in Glioblastoma [85]	Withdrawn	Pembrolizumab, Abemaciclib	2	0	1 December 2021
Pembrolizumab	NCT04977375	Trial of Anti-PD-1 Immunotherapy and Stereotactic Radiation in Patients with Recurrent Glioblastoma [86]	Recruiting	Pembrolizumab, Radiation therapy	1/2	10	9 December 2021
Pembrolizumab	NCT05084430	Study of Pembrolizumab and M032 (NSC 733972) [87]	Recruiting	M032, Pembrolizumab	1/2	28	25 February 2022
Pembrolizumab	NCT05235737	The Assessment of Immune Response in Newly Diagnosed Glioblastoma Patients Treated with Pembrolizumab [88]	Recruiting	Pembrolizumab	4	36	1 March 2022
Pembrolizumab	NCT05589961	Safety and Efficacy of TRPP Therapy in Glioblastoma Multiforme [89]	Recruiting	Temozolomide, Radiation therapy, Pembrolizumab	1	10	1 October 2022
Pembrolizumab	NCT05463848	Surgical Pembrolizumab +/− Olaparib with Temozolomide for recurrent Glioblastoma Multiforme [90]	Recruiting	Pembrolizumab, Olaparib, Temozolomide	2	78	21 October 2022
Pembrolizumab	NCT05700955	Neoadjuvant Chemoimmunotherapy in Recurrent Glioblastoma [91]	Recruiting	Pembrolizumab, Temozolomide	1	30	1 November 2022
Pembrolizumab	NCT05465954	Efineptakin Alfa and Pembrolizumab for the Treatment of Recurrent Glioblastoma [92]	Recruiting	Efineptakin alfa, Pembrolizumab	2	34	20 January 2023
Pembrolizumab	NCT06047379	Safety and Efficacy of NEO212 in Patients with Astrocytoma IDH-mutant, Glioblastoma IDH-wildtype or Brain Metastasis [56]	Recruiting	NEO212, Ipilimumab, Pembrolizumab, Nivolumab, Regorafenib, Carboplatin, Paclitaxel, FOLFIRI, Bevacizumab	1/2	134	1 November 2023
Pembrolizumab	NCT05973903	Lenvatinib, Pembrolizumab, and Tumor Treating Fields (TTF) for Second-line Treatment of Glioblastoma [93]	Not yet recruiting	Lenvatinib, Pembrolizumab, Tumor Treating Fields	1/2	47	1 January 2024
Pembrolizumab	NCT06157541	T Cells and Pembrolizumab for Recurrent and Newly Diagnosed Glioblastoma [94]	Recruiting	Cytomegalovirus-specific T cells, Pembrolizumab	1/2	58	8 February 2024
Pembrolizumab	NCT05879120	Randomized Study of Neo-adjuvant and Adjuvant Pembrolizumab with and without Targeted Blood-Brain Barrier Opening Using Exablate MRI-guided Focused Ultrasound (Exablate MRgFUS) for Recurrent Glioblastoma [95]	Not yet recruiting	Pembrolizumab, Exablate MRgFUS	2	10	30 July 2024
Pembrolizumab	NCT03311542	Expanded Access for Pembrolizumab (MK-3475) [96]	No longer available	Pembrolizumab	N/A	N/A	N/A
Avelumab	NCT03047473	Avelumab in Patients with Newly Diagnosed Glioblastoma Multiforme [97]	Completed	Avelumab	2	30	10 March 2017
Avelumab	NCT02968940	Avelumab with Hypofractionated Radiation Therapy in Adults with Isocitrate Dehydrogenase (IDH) Mutant Glioblastoma [98]	Completed	Avelumab, Radiation therapy	2	6	17 March 2017
Avelumab	NCT03291314	Clinical Trial on the Combination of Avelumab and Axitinib for the Treatment of Patients with Recurrent Glioblastoma [99]	Completed	Axitinib, Avelumab	2	52	3 May 2017
Avelumab	NCT03341806	Avelumab With Laser Interstitial Therapy for Recurrent Glioblastoma [100]	Completed	Avelumab, MRI-guided LITT	1	13	13 June 2018
Avelumab	NCT03750071	VXM01 Plus Avelumab Combination Study in Progressive Glioblastoma [101]	Active (not recruiting)	VXM01, Avelumab	1/2	30	21 November 2018
Durvalumab	NCT02336165	Phase 2 Study of Durvalumab (MEDI4736) in Patients with Glioblastoma [102]	Completed	Durvalumab, Radiotherapy, Bevacizumab	2	159	26 February 2015
Durvalumab	NCT02794883	Tremelimumab and Durvalumab in Combination or Alone in Treating Patients with Recurrent Malignant Glioma [103]	Completed	Durvalumab, Tremelimumab	2	36	1 November 2016
Durvalumab	NCT02866747	A Study Evaluating the Association of Hypofractionated Stereotactic Radiation Therapy and Durvalumab for Patients with Recurrent Glioblastoma [104]	Active (not recruiting)	Radiation Therapy, Durvalumab	1/2	108	17 January 2017
Durvalumab	NCT04521686	Study of LY3410738 Administered to Patients with Advanced Solid Tumors With IDH1 or IDH2 Mutations [105]	Active (not recruiting)	LY3410738, Gemcitabine, Cisplatin, Durvalumab	1	200	16 October 2020
Ipilimumab	NCT02017717	A Study of the Effectiveness and Safety of Nivolumab Compared to Bevacizumab and of Nivolumab with or Without Ipilimumab in Glioblastoma Patients [20]	Active (not recruiting)	Nivolumab, Bevacizumab, Ipilimumab	3	529	7 February 2014
Ipilimumab	NCT02311920	Ipilimumab and/or Nivolumab in Combination with Temozolomide in Treating Patients with Newly Diagnosed Glioblastoma or Gliosarcoma [23]	Completed	Ipilimumab, Nivolumab, Temozolomide	1	32	16 April 2015
Ipilimumab	NCT02794883	Tremelimumab and Durvalumab in Combination or Alone in Treating Patients with Recurrent Malignant Glioma [103]	Completed	Durvalumab, Tremelimumab	2	36	1 November 2016
Ipilimumab	NCT03233152	Intra-tumoral Ipilimumab Plus Intravenous Nivolumab Following the Resection of Recurrent Glioblastoma [30]	Unknown	Ipilimumab, Nivolumab	1	110	17 November 2016
Ipilimumab	NCT03879512	Autologous Dendritic Cells, Metronomic Cyclophosphamide and Checkpoint Blockade in Children with Relapsed HGG [31]	Recruiting	Dendric cells, Cyclophosphamide, Ipilimumab	1/2	25	7 February 2018
Ipilimumab	NCT03367715	Nivolumab, Ipilimumab, and Short-course Radiotherapy in Adults with Newly Diagnosed, MGMT Unmethylated Glioblastoma [32]	Completed	Nivolumab, Ipilimumab, Radiation therapy	2	10	7 February 2018
Ipilimumab	NCT03493932	Cytokine Microdialysis for Real-Time Immune Monitoring in Glioblastoma Patients Undergoing Checkpoint Blockade [37]	Completed	Nivolumab, BMS-986016	1	21	24 September 2018
Ipilimumab	NCT03422094	Neoantigen-based Personalized Vaccine Combined with Immune Checkpoint Blockade Therapy in Patients with Newly Diagnosed, Unmethylated Glioblastoma [39]	Terminated	NeoVax, Nivolumab, Ipilimumab	1	3	31 October 2018
Ipilimumab	NCT03430791	Trial of Combination Tumor Treating Fields (TTF; Optune), Nivolumab Plus/Minus Ipilimumab for Recurrent Glioblastoma [42]	Terminated	Nivolumab, Ipilimumab, NovoTTF200A (Optune)	2	5	5 December 2018
Ipilimumab	NCT03707457	Biomarker-Driven Therapy Using Immune Activators with Nivolumab in Patients with First Recurrence of Glioblastoma [43]	Terminated	Nivolumab, Anti-GITR Monoclonal Antibody MK-4166, IDO1 inhibitor INCB024360, Ipilimumab	1	3	22 March 2019
Ipilimumab	NCT04003649	IL13Ra2-CAR T Cells with or Without Nivolumab and Ipilimumab in Treating Patients with GBM [47]	Recruiting	IL13Ralpha2-CAR T cells, Ipilimumab, Nivolumab	1	60	2 December 2019
Ipilimumab	NCT04396860	Testing the Use of the Immunotherapy Drugs Ipilimumab and Nivolumab Plus Radiation Therapy Compared to the Usual Treatment (Temozolomide and Radiation Therapy) for Newly Diagnosed MGMT Unmethylated Glioblastoma [49]	Active (not recruiting)	Ipilimumab, Nivolumab, NovoTTF-100A, Radiation therapy, Temozolomide	2/3	147	1 September 2020
Ipilimumab	NCT04145115	A Study Testing the Effect of Immunotherapy (Ipilimumab and Nivolumab) in Patients with Recurrent Glioma with Elevated Mutational Burden [51]	Suspended	Ipilimumab, Nivolumab	2	37	24 December 2020
Ipilimumab	NCT04606316	Surgical Nivolumab and Ipilimumab for Recurrent GBM [52]	Active (not recruiting)	Nivolumab, Placebo, Ipilimumab	1	63	1 February 2021
Ipilimumab	NCT04817254	Association of Peripheral Blood Immunologic Response to Therapeutic Response to Adjuvant Treatment with Immune Checkpoint Inhibition (ICI) in Patients With Newly Diagnosed Glioblastoma or Gliosarcoma [54]	Recruiting	Temozolomide, Ipilimumab Nivolumab	2	58	8 December 2021
Ipilimumab	NCT05074992	A Trial of Neoadjuvant Therapy in Patients with Newly Diagnosed Glioblastoma [106]	Terminated	Ipilimumab	2	1	24 August 2022
Ipilimumab	NCT06047379	Safety and Efficacy of NEO212 in Patients with Astrocytoma IDH-mutant, Glioblastoma IDH-wildtype or Brain Metastasis [56]	Recruiting	NEO212, Ipilimumab, Pembrolizumab, Nivolumab. Regorafenib, Carboplatin, Paclitaxel, FOLFIRI, Bevacizumab	1/2	134	1 November 2023
Ipilimumab	NCT06097975	A Clinical Trial on Combined (Neo-)Adjuvant Intravenous Plus Intracranial Administration of Ipilimumab and Nivolumab in Recurrent Glioblastoma [57]	Not yet recruiting	Nivolumab, Ipilimumab	1	18	1 January 2024
Ipilimumab	NCT03460782	An Expanded Access Program of Ipilimumab for Patients with Glioblastomas and Gliomas [107]	No longer available	Ipilimumab	N/A	N/A	N/A
Cemiplimab	NCT03491683	INO-5401 and INO-9012 Delivered by Electroporation (EP) in Combination with Cemiplimab (REGN2810) in Newly Diagnosed Glioblastoma (GBM) [108]	Active (not recruiting)	INO-5401, INO-9012, Cemiplimab, Radiation Therapy, Temozolomide	1/2	52	31 May 2018
Cemiplimab	NCT04006119	Study of Ad-RTS-hIL-12 + Veledimex in Combination with Cemiplimab in Subjects with Recurrent or Progressive Glioblastoma [109]	Completed	Ad-RTS-hIL-12, Veledimex, Cemiplimab	2	40	1 August 2019
Cemiplimab	NCT04826393	ASP8374 + Cemiplimab in Recurrent Glioma [110]	Active (not recruiting)	ASP8374, Cemiplimab	1	14	9 March 2022
Tislelizumab	NCT05502991	Sintilimab (One Anti-PD-1 Antibody) Plus Low-dose Bevacizumab for ctDNA-level-relapse and Clinical-relapse Glioblastoma [111]	Not yet recruiting	Tislelizumab, Bevacizumab	2	60	11 December 2022
Tislelizumab	NCT05811793	Efficacy and Safety of SCAI of Bevacizumab Combined with IC of Tislelizumab in the Treatment of Recurrent Glioblastoma [112]	Not yet recruiting	Tislelizumab, Bevacizumab	N/A	36	15 April 2023
Tislelizumab	NCT05540275	Tislelizumab (One Anti-PD-1 Antibody) Plus Low-dose Bevacizumab for Bevacizumab Refractory Recurrent Glioblastoma [113]	Not yet recruiting	Tislelizumab, Bevacizumab	2	30	5 October 2023
Tislelizumab	NCT06353360	TTF in Combination With TMZ and Tislelizumab in The Treatment of Newly Diagnosed Glioblastoma [114]	Not yet recruiting	TTF, Tislelizumab, Temozolomide	2	30	5 April 2024
Tremelimumab	NCT02794883	Tremelimumab and Durvalumab in Combination or Alone in Treating Patients with Recurrent Malignant Glioma [103]	Completed	Durvalumab, Tremelimumab	2	36	1 November 2016
Relatlimab	NCT02658981	Anti-LAG-3 Alone and in Combination w/ Nivolumab Treating Patients w/ Recurrent GBM (Anti-CD137 Arm Closed 10/16/18) [29]	Completed	Anti-LAG-3 Monoclonal Antibody BMS 986016, Nivolumab, Anti-CD137	1	63	24 August 2016
Relatlimab	NCT03493932	Cytokine Microdialysis for Real-Time Immune Monitoring in Glioblastoma Patients Undergoing Checkpoint Blockade [37]	Completed	Nivolumab, BMS-986016	1	21	24 September 2018
Relatlimab	NCT06325683	Anti-Lag-3 (Relatlinib) and Anti-PD-1 Blockade (Nivolumab) Versus Standard of Care (Lomustine) for the Treatment of Patients With Recurrent Glioblastoma [58]	Not yet recruiting	Lomustine, Nivolumab, Relatlimab	2	178	16 August 2024

**Table 2:** Phase 3 Trials of Immune Checkpoint Inhibitors for Glioblastoma.

Sponsor	Agent	NCT Number	Title	Phase	Progression Free Survival (PFS) (months)	Overall Survival (OS)(months)
National Cancer Institute	Nivolumab + Ipilimumab	NCT04396860 [49]	NRG-BN007: Testing the Use of the Immunotherapy Drugs Ipilimumab and Nivolumab Plus Radiation Therapy Compared to the Usual Treatment (Temozolomide and Radiation Therapy) for Newly Diagnosed MGMT Unmethylated Glioblastoma	2/3	Preliminary for phase 2: Radiation Therapy + Temozolomide: 8.5 (CI: 7.1 to 10.4) Radiation Therapy + Nivolumab + Ipilimumab: 7.7 (CI: 6.5 to 8.5)	Not reported yet
Bristol-Myers Squibb	Nivolumab	NCT02617589 [26]	CHECKMATE 498: An Investigational Immuno-Therapy Study of Nivolumab Compared to Temozolomide, Each Given With Radiation Therapy, for Newly-diagnosed Patients With Glioblastoma (GBM, a Malignant Brain Cancer)	3	Nivolumab + Radiation Therapy: 6.01 (CI: 5.65 to 6.21) Temozolomide + Radiation Therapy: 6.21 (CI: 5.91 to 6.74)	Nivolumab + Radiation Therapy: 13.40 (CI: 12.62 to 14.29) Temozolomide (TMZ) + Radiation Therapy: 14.88 (CI: 13.27 to 16.13)
Bristol-Myers Squibb	Nivolumab, Bevacizumab, Nivolumab + Ipilimumab	NCT02017717 [20]	CHECKMATE 143: A Study of the Effectiveness and Safety of Nivolumab Compared to Bevacizumab and of Nivolumab With or Without Ipilimumab in Glioblastoma Patients	3	Nivolumab: 1.51 (CI: 1.48 to 1.61) Bevacizumab: 3.61 (CI: 2.99 to 4.60)	Nivolumab: 7.8 (CI: 4.1 to 13.3) Bevacizumab: 23.1 (CI: 16.7 to 30.5)
Bristol-Myers Squibb	Nivolumab	NCT02667587 [27]	CHECKMATE 548: An Investigational Immuno-therapy Study of Temozolomide Plus Radiation Therapy With Nivolumab or Placebo for Newly Diagnosed Patients With Glioblastoma (GBM, a Malignant Brain Cancer)	3	Radiotherapy, Temozolomide + Nivolumab: 10.64 (CI: 8.90 to 11.79) Radiotherapy, Temozolomide + Placebo: 10.32 CI: (9.69 to 12.45)	Radiotherapy, Temozolomide + Nivolumab: 31.34 (CI: 28.62 to 34.76) Radiotherapy, Temozolomide + Placebo: 32.99 (CI:31.01 to 35.09)
